# Fatty acid profiles and flavour-related compounds of retorted Korean ginseng chicken soup (Samgyetang) affected by pre-treated black garlic extract

**DOI:** 10.5713/ab.21.0575

**Published:** 2022-05-02

**Authors:** Farouq Heidar Barido, Dicky Tri Utama, Yeong Jong Kim, Sung Ki Lee

**Affiliations:** 1 Department of Applied Animal Science, College of Animal Life Sciences, Kangwon National University, Chuncheon 24341, Korea; 2 Department of Animal Science, Faculty of Agriculture, Universitas Sebelas Maret, Surakarta 57126, Indonesia; 3 Department of Animal Product Technology, Faculty of Animal Husbandry, Universitas Padjadjaran, Sumedang 45363, Indonesia; 4 WS Co., Ltd., Wanju 25729, Korea

**Keywords:** Black Garlic Extract, Fatty Acid Profile, Flavour Compounds, Non-volatile, Retorted Chicken Soup, Volatile

## Abstract

**Objective:**

This study aimed to characterize the effect of pre-treated black garlic (BG) extracts addition into retorted Korean ginseng chicken soup (Samgyetang) on the fatty acid composition and flavour-related indexes.

**Methods:**

Four different treatments; Samgyetang made with a 5% (w/w) addition of garlic (G), fresh BG (FBG), oven-dried BG (DBG), or encapsulated BG (EBG) extracts were developed and compared to negative control (NC) without any extract addition. Prepared samples were cooked via retorting at 121.1°C, 1.5 kgf/cm^2^ for 1 h.

**Results:**

The BG treated samples were higher in C18:3n3 and C18:2n6 fatty acids, with thrombogenic index was 18% to 20% lower than the NC. EBG yielded the highest umami-related nucleotides (5′-guanosine monophosphate and 5′-inosine monophosphate) and modified some free amino acid (alyne, phenylalanine and leucine) thus possessed the highest equivalent umami concentration among samples. Some individual aldehydes (pentanal, hexanal, and heptanal) were lower, while furans and volatile sulfur compounds were higher than the NC and G treatment group, indicating a potential suppression of unpleasant flavour alongwith the intensificiation of favourable flavour from the addition of BG extracts into retorted Samgyetang.

**Conclusion:**

Taken together, the synergistic results of this study indicate that incorportating suitable pre-treatment of BG extract could be of critical importance for the development of the retorted Samgyetang with improved flavour and functionalities.

## INTRODUCTION

Coinciding with economic growth over recent decades, ready-to-eat (RTE) products have been widely consumed as part of an assiduous lifestyle to overcome the excessive time required for meal preparation [1,2]. Retort processing is the most common method adopted by the food industry to ensure the inactivation of pathogenic bacteria and to enable non-refrigerated storage of food products. Despite its advantageous effect on food sterilization, the discrepancies in its reported effect on the physicochemical characteristics and health aspects, particularly in meat products, have led to difficulties in establishing its advantages [3]. The most common effect of high-temperature and high-pressure cooking is the formation of lipid oxidation products (LOPs) followed by visual, nutritional, and flavour quality deterioration [4].

In Asian countries, chicken soup is one of the most consumed cuisines because of its ability to provide all the important nutrients required by the human body, including free amino acids (FAAs), polyunsaturated fatty acids (PUFAs), reducing sugars, and polysaccharides [5]. Korean ginseng chicken soup (Samgyetang), which is mainly prepared by boiling a whole chicken carcass stuffed with a mixture of glutinous rice, ginseng (*Panax ginseng*), and jujube (*Zhiziphus jujuba*), is a popular chicken soup, largely consumed as a health restorer during the summer season [2]. Its unique flavour characteristics, including meaty, fatty, and sweet flavours, are responsible for its high consumption. Synergistic interactions between non-volatile compounds, namely 5′-nucleotides, FAAs, and soluble sugars, together with volatile flavour compounds, such as 3-(methylthio) propanal, (E,E)-2,4-decadienal, methylpyrazine, 2-ethyl-4-methylthiazole, and 2-methylbutana, were assumed to strongly dictate the taste and flavour profile of chicken soup [5,6].

Considering appropriate food selection to play a more important role in extending life expectancy before physical and mental exercise [7], the number of health-conscious consumers worldwide has continuously increased over the past few years, as they are seeking both flavourful food and health improvements. This trend urges experimental studies to develop healthier food products by incorporating natural antioxidants. In meat products, natural antioxidants have been extensively studied, and polyphenols have been highlighted to play a major role in mitigating reactive oxygen species (ROS). Moreover, chicken meat, enriched with higher protein and PUFA levels and lower fat contents, is an affordable protein source that balances the daily nutrition required for humans [8]. However, undesirable changes after high-temperature cooking are primarily found in meat types with a high PUFA level, in which they are more susceptible to lipid oxidation [2,4].

Black garlic (BG) is a processed product of garlic (G; *Allium sativum*) with numerous antioxidant and therapeutic effects. Biochemical alterations of allicin and deoxidized alliin into sulfur-containing compounds, including S-allyl mercapto cysteine, S-allyl cysteine, diallyl sulfide, diallyl disulfide, diallyl trisulfide, and diallyl tetrasulfide, are generated after high temperature (60°C to 90°C) and humidity (60% to 80%) processing (21 to 72 days). These compounds have been extensively studied and are reported to be correlated with higher health-promoting effects compared to raw garlic (RG) [9,10]. Moreover, the absence of a pungent flavour from RG, which is instead converted into a sweet flavour, makes it preferable for general segmentations. Therefore, in recent years, BG is widely consumed in several regions, including North America, Europe, Asia-Pacific, South America, the Middle East, and Africa [11]. Considering its substantial functionalities, the addition of BG to RTE products, particularly chicken soup, has the potential to counteract LOPs and ROS during high-temperature processing, thus contributing to functional food while maintaining its optimum quality. However, as phenolic compounds are predominantly composed of unsaturated bonds that are readily damaged by oxidizing environments and high-temperature processing, pre-treatments are suggested to enhance their stability during high-temperature processing [12]. Therefore, the present study aimed to characterize the potential effect of pre-treatment of BG extracts prior to addition into retorted Samgyetang on the fatty acid composition and flavour profile variation.

## MATERIALS AND METHODS

### Preparation of black garlic extracts

**Figure 1:**
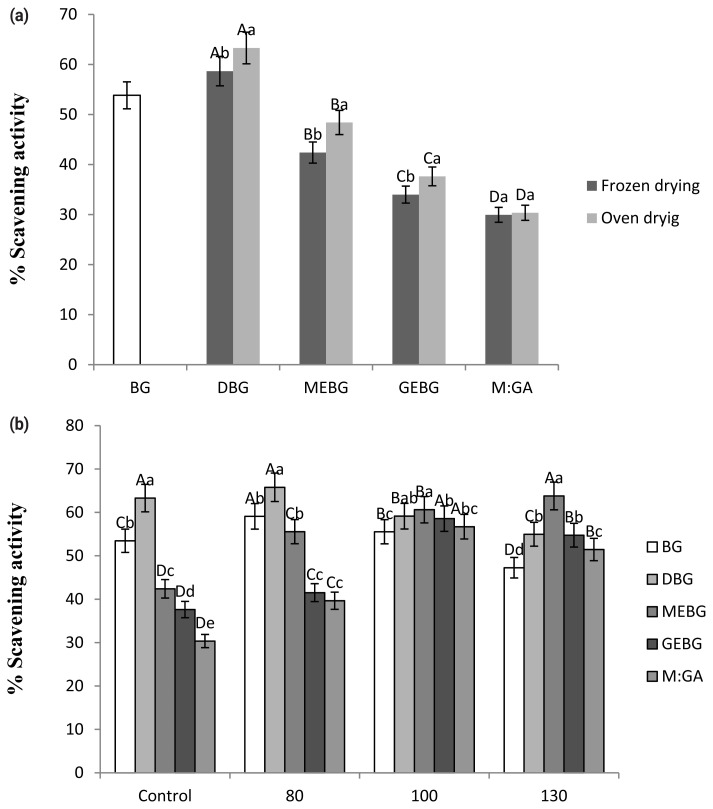
(a) Antioxidant activity of black garlic extract affected by drying and coating materials. (b) Antioxidant activity of the black garlic extract with diverse coating materials subjected to high temperature exposure. BG, black garlic extract; DBG, dried black garlic extract; MEBG, maltodextrin encapsulated black garlic extract; GBG, gum arabic encapsulated black garlic extract; M:GA, black garlic extract encapsulated with the mixture of maltodextrin and gum arabic. ^a,b^ Means with different letters within the same treatment group differ between drying methods (p<0.05). ^A–D^ Means with different letters among treatment groups differ significantly (p<0.05).

BG (moisture content: 66.70%±0.13%) was obtained from the local commercial industry Haena Food Co. (20160506929-1; Seoul, Korea). The extraction was performed according to the method described by Yuan et al [10] to obtain a phenolic solution. Initially, BG pre-treated in a dry base was mixed with 10 volumes of distilled water. The mixture was finely blended, heat extracted in a water bath at 80°C for 1 h, and subsequently filtered using Whatman filter paper number 1. The antioxidant activities, total phenolic and flavonoid contents, and moisture and pH values were compared, and their values are presented in Figure 1a. Concerning pre-treatment of BG, oven-drying and encapsulation with maltodextrin were chosen to compare its efficacy with that of RG and BG. Oven-drying treatment (180°C, 15 min) was chosen considering the preferred antioxidative profiles of oven-dried BG compared to that of freeze-dried BG (−70°C, 24 h). Encapsulation with maltodextrin was carried out owing to the vulnerability of phenolic extracts to high-temperature processing [12]. The addition of extract solution from various preparations of BG to Samgyetang was set at 5% (v/w) from the total weight, and the negative control (NC) was set with no addition of both G and BG extract.

### Sample preparation

The manufacturing procedure of Samgyetang employed retort cooking according to a previously reported protocol [1] with minor revisions. Five different treatments were prepared in triplicate, consisting of a NC (traditional ingredient stock), G (traditional ingredient stock + 5% G [v/w]), BG (traditional ingredient stock + 5% BG [v/w]), oven-dried black garlic (DBG; traditional ingredient stock + 5% DBG [v/w]), and encapsulated black garlic (EBG; traditional ingredient stock + 5% EBG [v/w]). The traditional ingredient stock was prepared by boiling distilled water together with herb pouches, which consisted of 5 g *Astragalus membranaceus* root, 8.5 g mulberry branch, 8 g *Kalopanax septemlobus* branch, 2 g licorice, and 9 g Siberian ginseng with 0.6% salt addition for 45 min. After placing skinless breast and thigh meat (commercial broiler, 200±10 g) into the retort pouch, 300 mL of prepared traditional ingredient stock and 100 mL of various G extract solutions were added, sealed, and cooked under retort conditions (121.1°C and 1.5 kgf/cm^2^ for 1 h).

### Antioxidant activity and total phenolic compound

The antioxidant activities of BG extract solution and Samgyetang prepared with various BG extracts were validated by using the diphenylpicrylhydrazyl (DPPH) assay. The reference method used was similar to that described by Islam et al [13]. The results were expressed as a scavenging percentage, and all analyses were performed in triplicate. The total phenolic content (TPC) were measured in triplicate at 765 nm and expressed as gallic acid equivalents (mg GAE/g sample), while the and total flavonoid content (TFC) was measured at 510 nm and expressed as catechin equivalents (mg CAE/g sample).

### Lipid oxidation

Malondialdehyde formation after retort processing was quantified using the 2-thiobarbituric acid reactive substances (TBARS) method. Chicken meat samples (0.5 g) were mixed with the antioxidant mixture (0.1 g), 3 mL of 1% tryptone bile agar in 0.3% NaOH, and 17 mL of 2.5% trichloroacetic acid in 36 mM HCl in a 25 mL TBARS test tube. The mixture was vortexed for 30 s prior to the addition of 2.5% trichloroacetic acid. After sealing, the tubes were heated in a water bath (BW-20G; JeoioTech, Yuseong, Daejeon, Korea) at 100°C for 30 min, and directly immersed in ice water to stop the reaction. Five mL of the aqueous sample was then mixed with 3 mL of chloroform in a 15 mL conical tube and centrifuged at 2,400×*g* for 30 min at 4°C (1248R; LaboGene, Lillerød, Denmark). The absorbance was recorded against a blank at 532 nm using a UV spectrophotometer (UV-mini 1240 PC; Shimadzu, Kyoto, Japan).

### Fatty acid composition

A gas chromatography (GC)/flame-ionization detection instrument (6890 N; Agilent Technologies, Santa Clara, CA, USA) with an autosampler (7683; Agilent Technologies, USA) was used to determine the fatty acid composition in the present study. Initially, finely ground samples (20 g) were extracted in duplicate according to Folch et al [14] with a chloroform-methanol (2:1 v/v) solution. Methylation to convert fatty acids into methyl esters was performed using 25% boron trifluoride in methanol at 80°C for 1 h. Fatty acid methyl esters were subsequently mixed with 1.5 mL hexane and 1 μL of the sample was injected into the GC port using an autosampler. The injector temperature was set at 250°C with a 100:1 split ratio. Fatty acid methyl esters were separated using a WCOT-fused silica capillary column (100 m×0.25 mm i.d., 0.20 μm film thickness; Varian Inc., Palo Alto, CA, USA) with a 1.0 mL/min helium flow. The detailed program of the oven was: 150°C/1 min, 150°C to 200°C at 7°C/min, 200°C/5 min, 200°C to 250°C at 5°C/min, and 250°C/10 min. The detector temperature was 275°C. Fatty acids were identified by comparing the identified peaks with the retention time of fatty acid standards (47,015-U; Sigma-Aldrich, St. Louis, MO, USA). The peak area of each identified fatty acid was used to calculate the proportion (%) of the total identified peak area.

### Free amino acid contents

The FAA was determined in triplicate according to the method of Jayasena et al [5] with slight modifications. Finely ground (500 mg) and 6 N HCl (20 mL) were placed into a 25 mL test tube. After being flushed with N_2_ gas for 30 s, a sealed tube was subjected to hydrolysis at 110°C for 16 h. Then, 100 μL of the hydrolyzed solution was evaporated under N2 gas for another 30 s, dissolved with 1 mL of Milli-Q water, vortexed, and the mixtures were filtered through a 0.45 μm PTFE filter. The FAA was quantified by high-performance liquid chromatography (HPLC) (Nexera X2 HPLC; Shimadzu, Kyoto, Japan) after derivatization with opthaldialdehyde 2 min prior to injection. The column size was 4.6×150 mm, with a 5-μm particle size, (Agilent Technologies, USA), and a 338 nm detection wavelength at 40°C. Mobile phase A was 40 mM NaH_2_PO_4_, pH 7.8, and mobile phase B was 45% acetonitrile, 45% methanol, and 10% Milli-Q water, with the separation performed at a 1.5 mL/min flow rate. The details of the gradient program were: 2% B for 0 and 0.5 min; 57% B for 20 min; 100% B for 20.1 and 23.5 min; 2% and 0% B for 23.6 and 25 min respectively; stop-time and post-time were 25 and 5 min, respectively.

### Volatile flavour compounds

A previous report by Akköse et al [15] was used to analyze volatile flavour compounds using GC/mass spectrometry (MS). Finely ground samples (5 g) were placed into a pre-heated 50 mL headspace vial at 60°C for 10 min. Carboxen/polydimethylsiloxane (CAR/PDMS; Sigma-Aldrich, USA) was used to extract the volatile flavour. Following extraction, the fibers were injected into the GC port set at 250°C and the volatiles were desorbed for 5 min at a 1:5 split ratio. After being subjected to GC, the volatile compounds were identified by MS, where the ion source temperature of the MS was set to 280°C with an electron impact of 70 eV. A standard library from the National Institute of Standards and Technology was used as a reference.

### Taste-related nucleotides

Quantification of the 5′-nucleotides, including adenosine monophosphate (AMP), inosine monophosphate (IMP), guanosine monophosphate (GMP), adenosine, and hypoxanthine, was performed using HPLC according to a previous study by Jayasena et al [5]. The mobile phase consisted of A 0.04% (v/v) triethylamine in phosphate buffer (58.72 mM Na_2_HPO_4_, 40 mM KH_2_PO_4_, pH 7.02 at 22°C), and B was a mixture of HPLC-grade distilled water and acetonitrile (40:60 v/v). The analysis was conducted in triplicate, wherein the absorbance was measured at 260 nm using diode array detectors, and identified peaks were compared with the retention time of the prepared standards (Sigma-Aldrich, USA).

### Statistical analysis

The data analyses in this study were performed using one-way analysis of variance using R version 3.6.1 (The R-foundation for Statistical Computing, Vienna, Austria). The significant mean value of each group following the treatment was continuously analyzed using Duncan’s multiple range test, with statistical significance set at p<0.05.

## RESULTS AND DISCUSSION

### Selection of Black garlic pre-treatment

The pre-treatment of BG, including drying and coating material selection, is expected to provide extensive protection on the quality and fatty acid composition during high-temperature processing. This optimization was performed through the measurement of antioxidant activity by a single factor experiment, and the results are displayed in Figure 1. As seen in Figure 1a, both drying methods exhibit a significantly higher antioxidant activity compared to fresh BG (FBG) by 8.90% and 17.40% for freeze-drying and oven-drying, respectively (p<0.05). However, BG extract that was previously subjected to oven drying had a significantly higher activity regardless of the coating material compared to that of frozen drying (p<0.05). A limited effect was observed for the antioxidant activity of the mixture of maltodextrin and gum arabic as coating materials. Thus, in this study, oven drying was selected as the drying method for the BG extract.

Different coating materials for encapsulation were studied, and Figure 1b presents the DPPH scavenging activity of the BG extracts at various pre-treatments and temperature exposures. The temperature exposure was set to 1 h as the average retorting time required. The antioxidant activities of different pre-treatments were compared to those of FBG, according to their free radical scavenging activity. In FBG and DBG, the antioxidant activity reached its peak at 80°C and gradually degraded as the temperature increased (p<0.05). Meanwhile, regardless of the coating materials, the antioxidant activity of the encapsulated groups remained stable at 100°C, then gradually decreased after 130°C, except for the maltodextrin EBG (EBG) group. The highest antioxidant activity with 63.78% scavenging activity at 130°C was observed when maltodextrin was used as a coating material. Therefore, we selected maltodextrin as a coating material for BG extract.

### Antioxidant activity, total phenolic acid, and lipid oxidation

The antioxidant activity and TPC of the meat samples were significantly affected by high-temperature processing. As shown in Table 1, the highest antioxidant activity of the breast meat sample is observed at 60.22% when EBG extract is added. The order of antioxidant activity from high to low was EBG, DBG, FBG, G, and NC (p<0.05). Moreover, the total polyphenol and flavonoid contents of the breast samples were measured, and the results showed that FBG had the highest TFC, followed by EBG, DBG, G, and NC (p<0.05). Conversely, DBG-and EBG-treated groups had the highest TPC following exposure to retort processing at 28.77 and 29.79 GAE mg/100 mg, respectively. Meanwhile, the TPC for FBG, G, and the NC was 22.62, 18.51, and 12.36 mg/100 mg, respectively. Furthermore, regardless of pre-treatment, BG extract addition into retorted Samgyetang generated breast meat with a significantly higher TPC than that with G.

The formation of malondialdehyde (MDA) content in retorted Samgyetang decreased with the addition of G or BG extracts compared to that with no addition (NC, 1.51; G, 1.19; BG, 1.09; DBG, 0.95, and EBG, 0.61 MDA mg/kg). However, the strongest inhibition of MDA formation was found in the EBG-treated group, followed by DBG, FBG, G, and the NC (p<0.05). Additionally, in the comparison between FBG and DBG, DBG was more effective in reducing the concentration of LOPs. However, FBG was superior to G extract in protecting against lipid oxidation (p<0.05). The results of this study confirmed those of a previous study [16], in which BG extract exhibited a better suppressive activity on free radical and LOPs, including MDA. The altered phenolic compounds during BG processing are believed to be responsible for the superior suppression activity by neutralizing free radicals and chelating the metal ion [17]. Therefore, although G addition into meat products has been reported to decrease LOP formation [18], the higher phenolic concentration and antioxidant activity of the BG extract made it more effective in reducing the lipid oxidation rate, causing quality deterioration. Furthermore, the high antioxidant activity of the EBG group might be due to the extensive protection of antioxidant compounds during high-temperature exposure. Examined through the microscopic approach, maltodextrin as a coating material could exhibit a spherical form with a size of 30 to 50 μm that enables phenolic compound absorption and remains inside after the drying process. Therefore, the damage to coated phenolic compounds caused by high temperatures and oxidizing environments can be delayed [12].

### Fatty acid composition

Table 2 shows the fatty acid composition of the retorted Samgyetang following addition of G and various BG extracts. The predominant fatty acids observed in the NC were oleic acid (C18:1n9), palmitic acid (C16:0), linoleic acid (C18:2n6), stearic acid (C18:0), and palmitoleic acid (C16:1). The incorporation of G and BG extracts at various pre-treatments into retorted Samgyetang significantly modified the PUFA profiles, primarily through the alteration of linoleic acid and α-linolenic acid (C18:3n3). Extensive protection of the individual fatty acid, such as linoleic acid was also observed in the BG-treated groups regardless of pre-treatment, and thus, had a significantly higher value compared to the NC. Similarly, the proportion of C18:3n3 as the only n-3 fatty acid detected in this study was higher in the BG-treated groups than in the NC. Pittman et al [19] reported that various individual fatty acids hold important flavour properties. The docosahexaenoic, arachidonic, and oleic acid contribute for sweet, umami, and salty taste, while the linoleic acid has strong savoury and sour properties. Therefore, the extensive protection on the linoleic acid following treatment with the BG extracts may help to generate a richer savoury taste perception of the Samgyetang.

No statistical difference between G-and BG-treated groups was observed in the present study on the fatty acid composition of the retorted Samgyetang (p>0.05). Considering the saturated fatty acids (SFA) and monounsaturated fatty acids (MUFA), a slight variation was observed for C16:0 and C16:1 fatty acids, in which the NC had the highest percentage compared to that of G-and BG-treated groups (p<0.05). However, these differences did not significantly influence the SFA and MUFA percentages. The results of fatty acid composition observed in this study were in contrast with those of Mancini et al [20], who did not observe a significant alteration in rabbit patties following addition of G powder. The low percentage of G powder added was assumed to insignificantly modify the fatty acid composition. Thus, in this study, we proposed a higher amount of G and BG extracts added to meat products and revealed fatty alterations after addition at 5%. Indeed, during high-temperature processing, the generation of iron ions to promote lipid autoxidation is higher owing to the denaturation of heme proteins within the muscle. As a result, the formed free radicals would interact with unsaturated chain fatty acids and cause their continued degradation following high-temperature processing [21]. Antioxidant activity was previously reported to be capable of adjusting the fatty acid composition via free radical and iron ion scavenging activities [17]. The high antioxidant activity of BG extracts confirmed these mechanisms. Besides, based on the results, the inclusion of BG at any given condition may potentially contribute for the flavour improvement of Samgyetang.

### Volatile flavour compounds

Meat flavour is composed of various complex compounds, including aldehydes, furans, alcohols, hydrocarbons, ketones, and sulfur-containing substances, and its formation is highly influenced by either the Maillard reaction or macromolecule oxidation [22]. Using GC/MS, this study identified 34 volatile compounds in each chicken meat sample, as shown in Table 3. All identified compounds were grouped into aldehydes, alcohols, ketones, hydrocarbons, furans, esters, and volatile sulfur compounds. In the NC without the addition of G and BG extracts, the predominant compounds recognized by normalized relative intensity score 4.04 to 0.50 were hexanal (grass, tallow, fat), (E)-2-hexenal (green, leaf), heptanal (fat, citrus, rancid), (E)-2-octenal (green leaf, walnut), nonanal (fat, citrus, green), (Z)-4-decenal (green, must), decanal (orange peel, soap, tallow), (E)-2-decenal (tallow), 1-pentanol (fruit, balsamic), 1-octen-3-ol (mushrooms), 2-methylfuran (roasted meat), 2-pentylfuran (green bean, butter), and benzothiazole (cooked, meat, nutty, sulfur). The predominant compounds identified in G-treated samples were similar to those of the NC, namely octanal (fat, soap, lemon, green), 1-octen-3-ol, octane (alkane), 2,5-dimethylfuran (bacon, meaty, gravy, roasted), carbondisulfide (pleasant, sweet, ether like), benzothiazole, diallyl disulfide (garlic, sulphur), and pentyl formate (punget). Distinct differences were observed in the lower scores of pentanal (almond, malt, pungent), hexanal, and heptanal, which highly correlated with the rancid and unpreferable flavour [23] as a result of BG extract addition, regardless of pre-treatment. Qi et al [24] explained that among volatile organic compounds, aldehyde classes are categorized to having low odor threshold, thus even at low concentration, they may notably impart meat flavours. The heptanal, pentanal and hexanal of the aldehydes are mentioned to exihibit unpleasant perception, while the octanal and nonal are the pleasant substances. The 4,5-dimethyl-2-pentyl-3-oxazoline is the main product of the hexanal and its excessive concentration caused the rancid, pungent perception [25]. Accordingly, the hexanal was mentioned as one of the predominant compounds in cooked meat that positively correlated with TBARS formation and negatively correlated with the overall flavour preference [24]. Following treatment with the addition of BG extracts at any given condition, the hexanal, heptanal alongwith pentanal were significantly lower in quantity in comparison to that of NC and G treatment group. The concentration of pentanal was even the lowest when samgyetang incorporated with EBG. These results indicated a potential suppression of unpleasant flavour from the addition of BG extracts into retorted Samgyetang.

As explained by previous study [22], the Maillard reaction strongly contributes to meat flavour development. The production of pleasant or undesirable flavours depends on the interaction between amino acids and sugars. In the presence of heat, the interaction between cysteine and glucose tends to generate increased furans and pyrazines. However, when heat is absent, or during storage, the interactions between these compounds tend to produce sulfur compounds. As observed in the present study, high-temperature and high-pressure cooking via retorting enables the activation of the Schiff base, Strecker degradation, or other pathways, thus leading to the non-enzymatic melanoidins reaction and production of furans as the predominant compounds. Interestingly, the concentrations of volatile sulfur compounds and furans as end products were higher in the presence of BG extracts (p<0.05). The high concentration of cysteine as a result of aging G into BG is assumed to be responsible for this phenomenon. Under high-temperature cooking, cysteine and ribose are converted into 2-methyl-3-furanthiol, which is the major substance for the development of disulfides and thiols [26]; thus, the volatile sulfur compounds and furans were intensified. A significantly improved pleasant flavour of volatile sulfur compounds and furans compared to that of the NC (p<0.05). Supported by the performed studies that revealed the flavour intensification in processed chicken meat is strongly determined by sulfur-and nitrogen-containing compounds, rather than by hydrocarbons [2,23,26].

### Free amino acid contents

The incorporation of BG extract into retorted Samgyetang had a minor effect on the FAA concentration, as shown in Table 4. Glutamic acid, serine, alanine, and lysine were the most predominant FAAs identified in this study. The total FAA concentration in the NC was recorded at 167.87 mg/100 g, in accordance with previous reports in breast meat [27]. The total FAA concentration did not differ, even after the addition of G and BG extracts (p>0.05). Meanwhile, the individual FAA concentration appeared to be affected by adding the BG extract, wherein, regardless of pre-treatment, increased alanine concentration was observed in BG-treated groups with 29.21, 28.05, and 29.09 mg/100 g for FBG, DBG, and EBG, respectively. Contrastingly, the addition of BG extracts to Samgyetang contributed to a significant decrease in leucine and phenylalanine in comparison with that of the NC and G extract-treated groups (p<0.05). No further differences were observed in this study for each FAA (p>0.05).

Studies have shown that the primary factor that determines FAA concentration in chicken meat products is the crude protein content of the raw material used [23,26,28]. Thus, the total FAA concentration of either native chicken or spent hen chicken was reported to be higher than that of commercial broilers because of the high protein content. Although the cooking process was performed at a high temperature, the total FAA concentration remained unchanged, which was consistent with a previous report [29]. Moreover, the incorporation of an additional ingredient might influence the FAA concentration in chicken meat products, in which the addition of potato mash contributed to the modified concentration of most flavour FAAs in spent hen chicken nuggets [23]. Contrarily, with regard to the modified FAA in this study, the flavour identity of the alanine is reported to impart a sweet and pleasant flavour. Moreover, in the presence of Na salt, alanine might exhibit a synergistic effect with IMP to generate an umami flavour [26,27]. This FAA has been reported to be responsible for the major sweet flavour in crab meat [30]. Meanwhile, phenylalanine and leucine are bitter and sulfur-categorized FAAs. The results of this study indicated that the addition of BG extract exhibited a sweeter flavour while maintaining a lower bitterness sensation in retorted Samgyetang, with the optimum effect observed via pre-treatment with encapsulation.

### Taste-related nucleotides

The 5′-nucleotides are the predominant compounds in chicken meat that contribute to the unique umami taste [5,22]. During cooking, these compounds may interact with FAAs and enhance the monosodium glutamate-like flavour of food products [5]. In this study, the profiles of 5′-nucleotides, including AMP, GMP, IMP, inosine, and hypoxanthine, were quantified, and the results are displayed in Table 5. Retorted Samgyetang supplemented with either G or BG extracts had a significantly higher IMP concentration than that of the NC. IMP concentration was the highest in the G-treated group (128.88 mg/100 g), followed by encapsulation (126.31 mg/100 g), FBG (125.84 mg/100 g), DBG (124.74 mg/100 g) and NC (121.44 mg/100 g). Concerning GMP, the highest concentration was observed at 12.01 mg/100 g in Samgyetang with added BG extract that was previously encapsulated with maltodextrin. In addition, the order of GMP concentration from high to low was FBG (9.56 mg/100 g), DBG (8.81 mg/100 g), G-treated (7.33 mg/100 g), and NC (6.19 mg/100 g). Furthermore, inosine was identified as the predominant nucleotide in this study. Its concentration was the highest among the investigated nucleotides and ranged from 193.33 to 254.45 mg/100 mg. The remaining nucleotides (AMP and hypoxanthine) were undetected in the present study.

The 5′-nucleotide compounds contribute differently to the flavour profile of meat. Studies have classified IMP as the most important nucleotide compound that imparts an umami flavour. GMP is also characterized to strongly enhance the umami and meaty flavours. However, the concentration of hypoxanthine is not expected to be high because of its association with bitterness [5,30]. The incorporation of BG extract that was previously encapsulated with maltodextrin into retorted Samgyetang was potentially advantageous for imparting a stronger umami flavour by exhibiting significantly higher concentrations of GMP and IMP, as shown in this study. In the NC, the IMP concentration tended to be higher (121.44 mg/100 g vs 94.40 mg/100 g) than in the previous study for commercial broilers [31]. Furthermore, the high concentration of inosine observed in this study is in accordance with a previous study on chicken meat [26]. High-temperature treatment considerably induced the degradation of IMP into inosine. This study also confirmed that the increased inosine concentration is concomitant with an increased rate of IMP degradation [5,23,26].

## CONCLUSION

In this study, we elucidated the effect of addition of various pre-treated BG extracts on the quality of retorted Samgyetang. The incorporation of BG extract was proven to extend the protective effect on certain PUFAs, including taste-related fatty acids; linoleic acid (C18:2n6) and α-linolenic acid (C18:3n3). Certain individual aldehydes (pentanal, hexanal, and heptanal) were lower, while the 2-methyl furan, carbon disulfide, and ethyl acetate were found to be higher following BG extract addition, with an apparent effect observed in retorted Samgyetang supplemented with EBG extract. These obtained data indicating a potential suppression of unpleasant flavour alongwith the intensificiation of favourable flavour from the addition of BG extracts into retorted Samgyetang. In addition, the EBG group also exhibited the highest equivalent umami concentration among treatments because of the higher GMP content and effect of FAAs (alanine, phenylalanine, and leucine). A higher antioxidant profile and lower LOP formation were observed following BG extract addition in comparison with that of the G extract and NC, with a distinct effect documented for the EBG group. The results of the present study indicated that incorporation of BG extract, particularly with that of pre-treated BG via maltodextrin encapsulation, might have significant effects on health and flavour improvement of the retorted Samgyetang.

## Figures and Tables

**Table 1:** Antioxidative status, total phenolic compounds and lipid oxidation of the retorted Samgyetang affected by pre-treated black garlic extracts

Variables	Treatments^1)^					SEM	p-value

	NC	G	FBG	DBG	EBG		
Antioxidant activity (%)	23.44^e^	35.28^d^	49.41^c^	56.32^b^	60.22^a^	1.75	<0.05
TPC (GAE mg/100 g)	12.36^d^	18.51^c^	22.62^b^	28.77^a^	29.79^a^	3.66	<0.05
TFC (CE mg/100 g)	0.30^e^	0.60^d^	1.40^a^	0.90^c^	1.30^b^	0.01	<0.05
TBARS (MDA mg/kg)	1.51^a^	1.19^b^	1.09^b^	0.95^c^	0.61^d^	0.08	<0.05

SEM, standard error of the mean; TPC, total phenolic compounds expressed as gallic acid equivalent mg/100 g; TFC, total flavonoid compounds expressed as catechin equivalent mg/100 g; TBARS, 2-thiobarbituric acid reactive substances; MDA, malondialdehyde.

1) NC, negative control or retorted chicken soup without black garlic extract addition; G, retorted chicken soup added with garlic extract; FBG, retorted chicken soup added with fresh black garlic; DBG, retorted chicken soup added with oven dried black garlic extract; EBG, retorted chicken soup added with maltodextrin encapsulated black garlic extract.

a–e Mean values within the same rows with the different superscripts are significantly different among treatments (p<0.05).

**Table 2:** Fatty acid composition of the retorted Samgyetang affected by pre-treated black garlic extracts

Fatty acid (%)	Treatments^1)^					SEM	p-value

	NC	G	FBG	DBG	EBG		
C14:0	1.05	1.20	1.16	1.10	1.36	0.21	0.37
C16:0	27.55^a^	25.77^ab^	25.31^ab^	25.43^ab^	24.89^b^	2.04	<0.05
C16:1	5.84^a^	4.96^b^	5.40^ab^	5.31^ab^	5.50^ab^	0.28	<0.05
C18:0	6.12	6.27	6.64	6.46	6.21	0.45	0.15
C18:1n9	40.56	40.79	39.77	40.07	39.96	1.48	0.07
C18:2n6	16.31^b^	17.49^ab^	18.13^a^	18.22^a^	18.26^a^	0.94	<0.05
C18:3n6	0.96	1.09	1.04	0.95	1.05	0.17	0.10
C18:3n3	0.82^b^	1.56^a^	1.78^a^	1.94^a^	1.89^a^	0.22	<0.05
C20:4n6	0.55	0.65	0.51	0.51	0.67	0.11	0.08
C22:4n6	0.24	0.22	0.26	0.21	0.21	0.01	0.06
SFA	34.72	33.24	33.11	32.99	33.46	1.38	0.13
MUFA	46.40	45.75	45.17	45.38	45.46	2.11	0.08
PUFA	18.88^b^	21.01^a^	21.72^a^	21.63^a^	21.08^a^	1.09	<0.05
AI	0.49	0.46	0.45	0.45	0.46	0.02	1.12
TI	0.50^a^	0.38^b^	0.44^b^	0.43^b^	0.42^b^	0.02	<0.05

SEM, standard error of the mean; SFA, saturated fatty acid; MUFA, monounsaturated fatty acids; PUFA, polyunsaturated fatty acids; AI, atherogenic index; TI, thrombogenic index.

1) NC, negative control or retorted chicken soup without black garlic extract addition; G, retorted chicken soup added with garlic extract; FBG, retorted chicken soup added with fresh black garlic; DBG, retorted chicken soup added with oven dried black garlic extract; EBG, retorted chicken soup added with maltodextrin encapsulated black garlic extract.

a,b Mean values within the same rows with the different superscripts are significantly different among treatments (p<0.05).

**Table 3:** Volatile flavor profile of the retorted Samgyetang affected by pre-treated black garlic extracts

Compounds (area×10^5^)	Treatments^1)^					SEM	p-value	Odor

	NC	G	FBG	DBG	EBG			
Aldehydes								
Pentanal	1.00^a^	0.62^b^	0.50^b^	0.40^b^	0.05^c^	0.11	<0.05	Almond, malt, pungent
Acetal	0.32	0.45	0.61	0.46	0.78	0.05	0.09	Fruit, cream, cabbage
Hexanal	1.49^a^	1.54^a^	0.65^b^	0.82^b^	0.76^b^	0.23	<0.05	Grass, tallow, fat
(E)-2-Hexenal	2.10	2.38	2.41	2.37	2.65	0.57	0.31	Green, leaf
Heptanal	1.68^a^	1.46^a^	0.86^b^	0.96^b^	0.84^b^	0.03	<0.05	Fat, citrus, rancid
Octanal	0.66	0.79	0.52	0.73	0.72	0.02	0.11	Fat, soap, lemon, green
(E)-2-octenal	0.54	0.86	0.86	0.57	1.05	0.20	0.09	Green leaf, walnut
Nonanal	2.38	2.61	2.46	2.26	2.84	0.52	0.38	Fat, citrus, green
(Z)-4-Decenal	2.47	2.36	2.42	2.18	2.08	0.46	0.28	Green, must
Decanal	0.50	0.55	0.52	0.51	0.53	0.04	1.06	Soap, orange peel, tallow
(E)-2-Decenal	0.99	1.03	1.13	0.90	1.21	0.18	1.18	Tallow
(E,E)-2,4-Decadienal	0.08	0.13	0.39	0.30	0.40	0.32	0.07	Seaweed
2-Butyl-2-octenal	0.22	0.21	0.48	0.28	0.40	0.25	0.24	Green, nut, fat
Butanal, 3-methyl	0.10	0.24	0.24	0.21	0.28	0.13	0.97	Aldehydic, fatty, malt, chocolate
Alcohols								
1-Pentanol	1.19	1.19	1.88	1.22	1.40	0.82	1.09	Fruit, balsamic
1-Octen-3-ol	0.88	0.94	0.99	0.85	0.93	0.05	0.75	Mushrooms, compound excreted by many insects
Ketones								
Acetoxyacetone	0.35	0.31	0.71	0.43	0.55	0.35	0.39	Green, flower, ether
2-Heptanone	0.05	0.06	0.07	0.03	0.08	0.06	0.88	Soap
Octane-2,5-dione	0.03	0.05	0.03	0.06	0.06	0.04	0.57	Green leaf, walnut
Hydrocarbons								
Octane	0.40	0.42	0.43	0.42	0.48	0.07	0.61	Alkane
Toluene	0.23	0.29	0.47	0.25	0.39	0.29	0.71	Paint
dodecane	0.09	0.05	0.05	0.04	0.05	0.05	0.21	Alkane
tridecane	0.04	0.05	0.05	0.04	0.05	0.01	1.05	Alkane
Furans								
2-Methylfuran	3.12^b^	3.22^b^	4.21^a^	4.03^a^	4.04^a^	0.09	<0.05	Roasted meat
2,5-Dimethylfuran	0.69	0.67	0.87	0.47	1.09	0.51	0.81	Bacon, meaty, gravy, roasted
Furan, 3-methyl	0.31^c^	0.55^b^	0.68^a^	0.59^ab^	0.71^a^	0.10	<0.05	Roasted meat
Volatile sulfur compound								
2-Pentylfuran	0.53^c^	0.91^b^	1.72^a^	1.68^a^	1.87^a^	0.25	<0.05	Green bean, butter
Carbondisulfide	0.28^c^	2.15^b^	3.53^a^	3.23^a^	3.90^a^	0.71	<0.05	Pleasant, sweet, ether like
Benzothiazole	0.95	0.81	0.88	0.87	0.95	0.05	0.08	Gasoline, coffee, cooked, meat, nutty, sulfur
Diallyl disulphide	0.46^b^	0.82^a^	0.45^b^	0.47^b^	0.44^b^	0.09	<0.05	Garlic, sulphur
Ethyl acetate	0.19^c^	0.74^b^	0.92^a^	0.83^a^	1.07^a^	0.33	<0.05	Pinneapple
Pentyl formate	0.30	0.19	0.09	0.11	0.12	0.27	0.23	Pungent
Pentyl hexanoate	0.43	0.21	0.23	0.42	0.46	0.19	0.09	Apple peel, fruit
Hexyl hexanoate	0.28	0.34	0.47	0.27	0.44	0.31	0.51	Apple peel, peach

SEM, standard error of the mean.

1) NC, negative control or retorted chicken soup without black garlic extract addition; G, retorted chicken soup added with garlic extract; FBG, retorted chicken soup added with fresh black garlic; DBG, retorted chicken soup added with oven dried black garlic extract; EBG, retorted chicken soup added with maltodextrin encapsulated black garlic extract.

a–c Mean values within the same rows with the different superscripts are significantly different among treatments (p<0.05).

**Table 4:** Free amino acid composition (FAA) of the retorted Samgyetang affected by pre-treated black garlic extracts

FAA (μg/mL)	Taste threshold	Treatments^1)^					SEM	p-value

		NC	G	FBG	DBG	EBG		
Aspartic acid (Asp)	100	17.18	18.05	17.92	17.05	18.20	1.07	0.11
Threonine (Thr)	260	11.21	12.01	12.17	11.98	12.24	0.92	0.09
Serine (Ser)	150	24.08	23.99	25.19	24.77	25.02	0.87	0.21
Glutamic acid (Glu)	30	30.11	31.01	30.87	30.75	30.97	0.62	1.21
Glycine (Gly)	130	18.24	18.43	17.92	17.86	18.05	0.79	0.28
Alanine (Ala)	60	27.24^b^	27.12^b^	29.09^a^	28.05^ab^	29.21^a^	0.82	<0.05
Cysteine (Cys)	-	nd	nd	nd	nd	nd	nd	nd
Valine (Val)	40	3.17	3.21	3.03	3.10	3.15	0.07	1.02
Methionine (Met)	30	1.76	1.81	1.80	1.82	1.77	0.09	0.74
Isoleucine (Ile)	90	2.16	2.14	2.19	2.11	2.10	0.03	0.81
Leucine (Leu)	190	5.02^a^	4.98^a^	4.54^ab^	4.81^a^	3.97^b^	0.36	<0.05
Tyrosine (Tyr)	-	1.18	1.10	1.22	1.23	1.14	0.04	0.26
Phenylalanine (Phe)	90	4.15^a^	4.07^a^	2.17^b^	2.97^ab^	2.08^b^	1.05	<0.05
Lysine (Lys)	50	20.43	20.16	20.27	20.22	21.08	0.97	0.15
Arginine (Arg)	50	8.07	8.25	7.96	8.11	8.18	0.21	0.32
Proline (Pro)	300	3.04	3.09	3.11	3.01	3.01	0.11	1.05
Total FAA	-	167.87	170.37	172.86	170.06	174.00	5.77	0.07

SEM, standard error of the mean; nd, not detected.

1) NC, negative control or retorted chicken soup without black garlic extract addition; G, retorted chicken soup added with garlic extract; FBG, retorted chicken soup added with fresh black garlic; DBG, retorted chicken soup added with oven dried black garlic extract; EBG, retorted chicken soup added with maltodextrin encapsulated black garlic extract.

a,b Mean values within the same rows with the different superscripts are significantly different among treatments (p<0.05).

**Table 5:** Taste-related nucleotides and equivalent umami concentration of the retorted Samgyetang affected by pre-treated black garlic extracts

Variables (μg/mL)	Treatments^1)^					SEM	p-value

	NC	G	FBG	DBG	EBG		
GMP	6.19^e^	7.33^d^	9.56^b^	8.81^c^	12.01^a^	0.54	<0.05
IMP	125.84^ab^	128.88^a^	124.74^b^	121.44^c^	126.31^ab^	0.75	<0.05
Inosine	228.83^c^	240.74^b^	254.45^a^	238.65^b^	193.33^d^	5.58	<0.05
AMP	nd	nd	nd	nd	nd	nd	nd
Hx	nd	nd	nd	nd	nd	nd	nd
EUC	2,651.86^b^	2,884.66^a^	2,960.28^a^	2,651.50^b^	3,027.21^a^	13.17	<0.05

SEM, standard error of the mean; nd, not detected; GMP, guanosine monophosphate; IMP, inosine monophosphate; AMP, adenosine monophosphate; Hx, hypoxanthine; EUC, equivalent umami concentration.

1) NC, negative control or retorted chicken soup without black garlic extract addition; G, retorted chicken soup added with garlic extract; FBG, retorted chicken soup added with fresh black garlic; DBG, retorted chicken soup added with oven dried black garlic extract; EBG, retorted chicken soup added with maltodextrin encapsulated black garlic extract.

a–e Mean values within the same rows with the different superscripts are significantly different among treatments (p<0.05).
